# Characteristics of low acuity prehospital emergency patients with 48-h mortality, an observational cohort study

**DOI:** 10.1186/s13049-022-01048-8

**Published:** 2022-12-08

**Authors:** Jesper A. Dyhring Petersen, Stig Nikolaj Blomberg, Freddy Lippert, Helle Collatz Christensen

**Affiliations:** 1 Department of Clinical Medicine, Faculty of Health and Medical Sciences, University of Copenhagen, Copenhagen, Denmark; 2 Copenhagen Emergency Medical Services, Copenhagen, Denmark; 3 Danish Clinical Quality Program (RKKP), National Clinical Registries, Copenhagen, Denmark

**Keywords:** EMS, Epidemiology, Triage

## Abstract

**Background:**

Every year an emergency medical technician or paramedic treats and transports up to several hundred patients. Only some patients are acutely seriously ill, and a few of these show only discrete signs and symptoms of their condition. This study aims to describe patients who died within 48 h of being admitted non-emergently to hospital by ambulance, examine the extent to which critically ill patients are recognized prehospitally, and identify clinical warning signs that might be frequently overlooked.

**Method:**

Registry based follow-up study on patients receiving an ambulance from the Copenhagen EMS in 2018. Data was included regarding the dispatch of the ambulance from the emergency services disposition system, ICD-10 hospital admission diagnoses from the National Patient Register, 48-h mortality from the Central Person Register and assessment and treatment in the ambulance by reviewing the electronic pre-hospital patient record.

**Results:**

In 2018 2279 patients died within 48 h after contact with the EMS, 435 cases met inclusion criteria. The patients’ median age was 83 years (IQR 75–90), and 374 (86.0%) had one or more underlying serious medical conditions. A triage category based on vitals and presentation was not assigned by the EMS in 286 (68.9%) cases, of which 38 (13.3%) would meet red and 126 (44.1%) orange criteria. For 409 (94.0%) patients, it was estimated that death within 48 h could not have been avoided prehospitally, and for 26 (6.0%) patients it was uncertain. We found 27 patients with acute aortic syndrome as admission diagnosis, of these nine (33.3%) had not been admitted urgently to a hospital with vascular surgery specialty.

**Conclusions:**

It was estimated that death within 48 h could generally not be avoided prehospitally. The patients’ median age was 83 years, and they often had serious comorbidity. Patients whose vital parameters met red or orange triage criteria were to a lesser degree triaged prehospitally, compared to patients in the yellow or green categories. Patients with acute aortic syndrome were not recognized by EMS 33.3% of the time.

**Supplementary Information:**

The online version contains supplementary material available at 10.1186/s13049-022-01048-8.

## Background

Over the last decades there has been an increasing specialization and centralizing of the hospital system in Denmark [1] with following longer distance to an emergency department for many citizens. In the event of serious illness or injury, the ambulance crews are now responsible for the patient for an increasing period of time. Physician staffed response vehicles have in varying extent been introduced in all the Danish regions, but they are only included in a fraction of all patient contacts [2].

Simultaneously the emergency medical technician (EMT) education in Denmark has undergone a substantial development, from in year 2000 consisting of only two courses of each five weeks duration, to now being an education lasting three years and seven months authorized by the National Board of Patient safety [3–6]. Furthermore, there has been introduced a supplementary paramedic (PM) course which provides additional medical and technical competencies.

Every year, an EMT treats and transports several hundred patients. Only a few patients are acutely seriously ill, and of these, some patients show only discrete signs and symptoms of their condition [7]. This entails the risk that a serious condition is missed by the ambulance crew, and definitive treatment delayed, with injury or death as a result.

Attempts have been made to prevent critical patients from being overlooked prehospitally e.g. by triage on the basis of vital values and presenting symptoms [8, 9].

Several studies examine the quality of handling and visitation of the emergency call itself at the regional Emergency Medical Communications Centers (EMCC) [10–12]. Others have described which patient groups brought to hospital by ambulance have the highest mortality by their admission diagnosis [13] and many studies focus on specific emergent conditions such as cardiac arrest, myocardial infarction, stroke, and dyspnea or special procedures or medications [7, 14–18].

No Danish studies have investigated the examination and treatment that took place prehospitally in the time between the emergency call and admission to hospital.

This study aims to describe patients who died within 48 h of being admitted non-emergently to hospital by ambulance, examine the extent to which critically ill patients are recognized prehospitally, and identify clinical warning signs that might be frequently overlooked.

## Method

This is a registry-based follow-up study on patients who were in contact with the ambulance service in the Region of Copenhagen in 2018. In Denmark every citizen has a unique Central Person Register number (CPR), which is used when in contact with public agencies and allows for cross referencing of data across all public registers [19, 20]. Data regarding the dispatch of the ambulance was acquired from the administrative dispatch system, assessment and treatment by the EMTs in the ambulance from the electronic Prehospital Patient Record (PPR) [21, 22], ICD-10 hospital admission diagnoses from the National Patient Register [23], and 48-h mortality from the CPR register.

### Setting

In Copenhagen, ambulances are dispatched through calls to the emergency call center (1–1–2), calls to the 1813-Medical Helpline or they are requisitioned by a third party e.g. hospitals, general practitioners, or the police.

All emergency calls to 1–1–2 for ambulance are received at the region's EMCC where it is answered by trained nurses or paramedics. For support and documentation of the conversation, they use the decision-making tool Danish Index for Emergency Care which is based on national guidelines for emergency treatment and is used by all EMCC’s in Denmark [24].

The 1813-Medical Helpline is for all non-life threatening and non-urgent calls regarding referral to an emergency department or medical advice outside of the patient’s GP’s opening hours. Calls to the 1813-Medical Helpline are handled by a nurse or physician trained in telephone consultation and they use a systematic visitation guide to determine what help is offered [25].

The Region of Copenhagen has 1.8 million inhabitants and ambulances were dispatched to 167,295 incidents in 2018. The ambulances in Copenhagen are staffed with EMTs or paramedics. There are five Mobile Critical Care Units (MCCU) [2] in the region staffed by an anesthesiologist and an assistant (EMT or PM), who are sent to critical patients, either immediately based on the telephone call, or at the request of the ambulance crew on site.

All prehospital patient contacts are documented by the ambulance crews in the PPR [21, 22], this includes assessment of the patient's present condition, medical history, measured vitals, treatment given to the patient, and visitation to hospital based on the same triage system used in-hospital [8, 9].

### Study population

All ambulance dispatches in Copenhagen in 2018 were included. Dispatches to patients where no Danish CPR number was registered were excluded. Patients who died within 48-h of contact with the EMS were identified by linking to the CPR register.

Additional exclusion criteria were: Interhospital transports, as there is no actual prehospital assessment of the patient. Ambulance calls without patient contact, e.g. where the patient was not found, or the call was cancelled. Ambulances sent as assistance to another unit, to avoid double registration of the individual patient contact. Patients under the age of 15, as children often present with different symptoms than adults. Patients who were brought to hospital with assigned priority A which is the highest assigned priority used for patients who are critically ill, as this indicates the ambulance crew had found the patient critically ill and unstable. Patients declared dead prehospitally. Patients in the terminal stage of a disease and/or with an already established ceiling of treatment.

Some patients had multiple contacts with the EMS within the 48 h, of these the last contact where the patient was treated on scene or taken to hospital alive was chosen.

### Data management

Primary investigator who is 5th year medical student with 20 years of experience as a paramedic evaluated all medical records. A check list was designed to ensure uniform evaluation of all incidents (See Additional file 1). For each incident it was registered how the ambulance crew had assessed the patient's clinical status on arrival and continuously afterwards, what treatment the patient had received and subsequent assigned triage category. Recorded warning signs and symptoms such as pain, seizures, dyspnea, and impaired consciousness as well as the time of onset was registered.

Vitals including respiratory rate, peripheral oxygen saturation, heart rate, blood pressure, level of consciousness (Glasgow Coma Scale), blood sugar and temperature were determined as being 'low', 'normal' or 'high' based on an overall assessment of the patient record and the patient's underlying medical conditions. If a triage category was not specified prehospitally, the triage category was recreated based on the recorded vitals. A protocol exists for calculating triagecategory (point 7 in Additional file 1). There are four categories (red, orange, yellow and green). Each category contains a set of condition regarding (A) airway, (B) breath, (C) circulation (D) Glasgow Coma Score and (E) temperature. All these vital signs are measured routinely and recorded in the PPR. For instance, to be triaged into the red category the patient must have either:

(A) threatened airway or stridor or
(B) SpO_2_ < 80% without O_2_ or SpO_2_ < 90% with O_2_ or RR > 35 or < 8 or
(C) HR > 140 or BPsys < 80 mmHg or
(D) Glasgow Coma Score ≤ 8 or
(E) Temperature < 32 °C.

Based on the call criteria in Danish Index [24] and notes in the PPR a working diagnosis was estimated, unless directly stated in the text. Subsequently, the working diagnosis was compared with the patient’s primary hospital admission diagnosis to see how they corresponded. It was assessed whether the patient was in a particular risk group or fragile such as suffering from dementia, nursing home resident, bedridden, wheelchair user, use of home oxygen, or long-term alcohol abuse, as these are indicators of increased risk of mortality [26].

Finally, based on the available information in the PPR and admittance diagnosis, it was assessed whether it could have been anticipated prehospitally that the patient was at risk of dying within 48 h and whether prehospital intervention could reduce the risk. The risk assessment (point 15–17 in Additional file 1) was performed in collaboration between a senior consultant with 20 + years of experience and the primary investigator applying his 20 years of experience as an ambulance paramedic. Death was deemed possibly preventable if the patient was either: (1) Triage level was not actively acknowledged (2) The apparent level of disease required admittance to a higher level hospital eg. Abdominal aneurism of the Aorta (3) Normal standard treatment protocols were not followed.

## Results

**Fig. 1:**
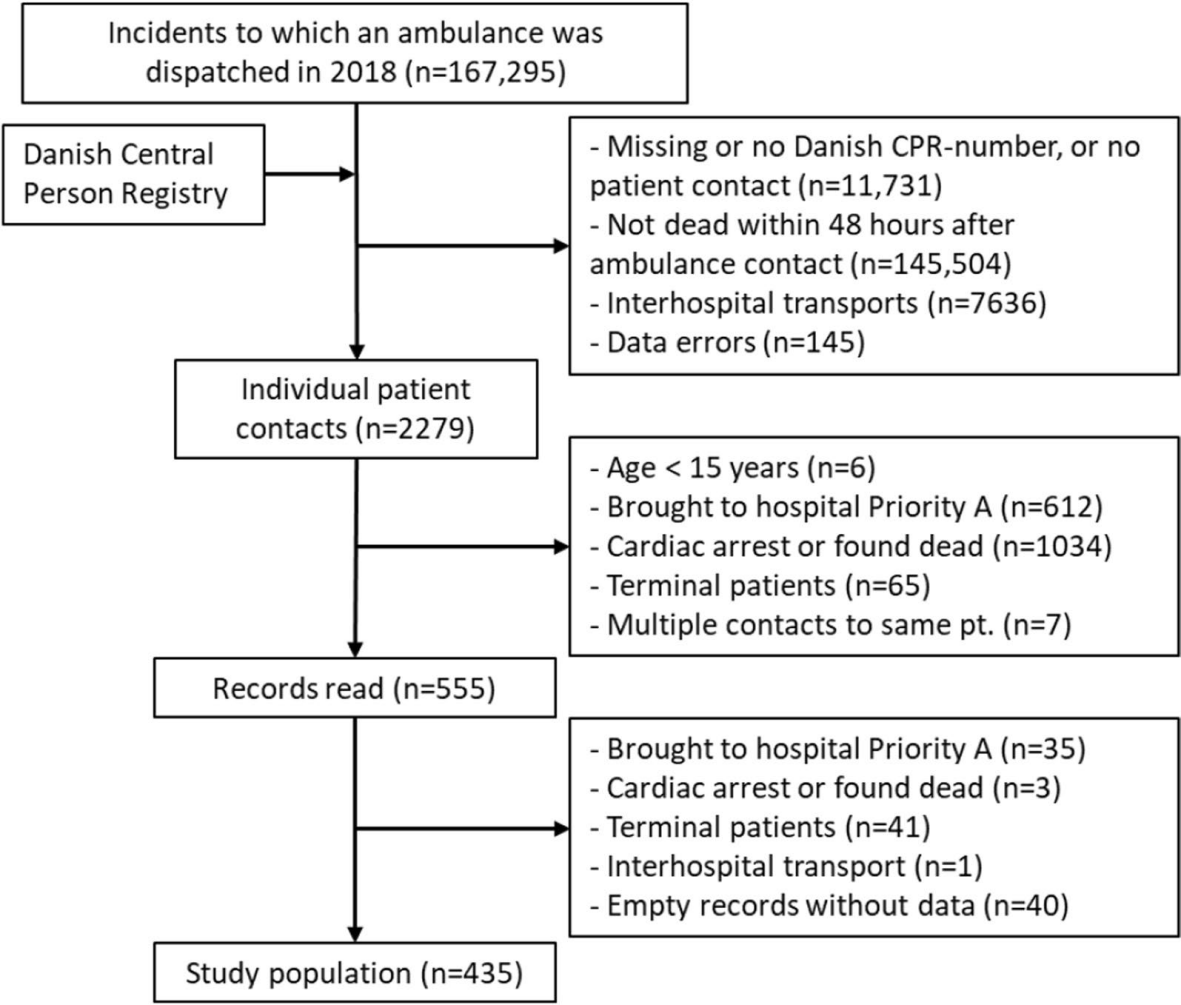
Study population, transports by Copenhagen Emergency Medical Services 2018

Ambulances were dispatched to a total of 167,295 incidents in Copenhagen in 2018. After exclusions 555 ambulance records were read of which 80 met prior exclusion criteria and 40 records contained no data. A total of 435 ambulance records were included in the study (Fig. 1).

**Fig. 2:**
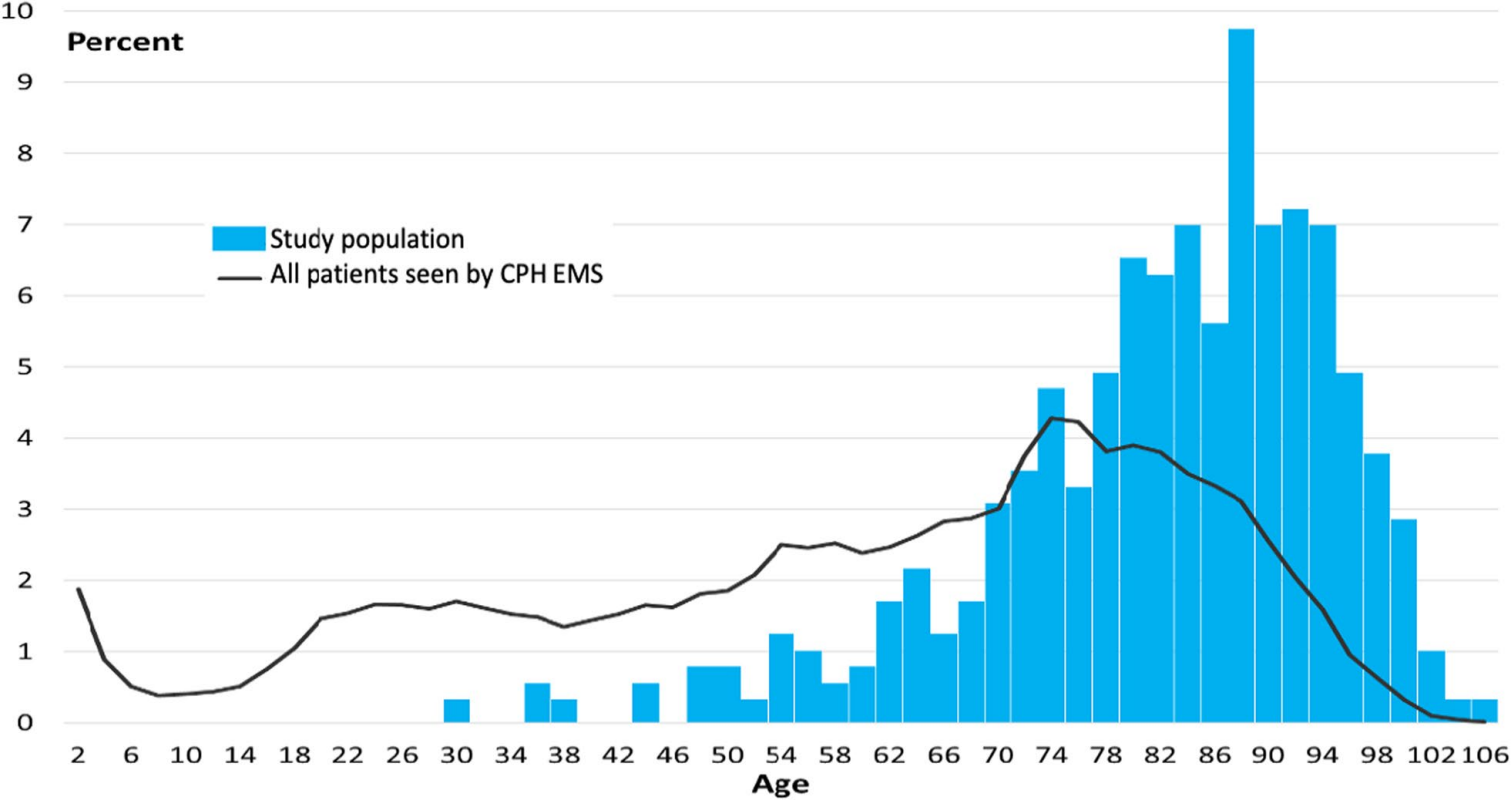
Age distribution by percentage of the study population and the overall population who received an ambulance in Copenhagen in 2018. The age distribution by percentage of the study population with 48-h mortality and who were brought non-emergently to hospital, compared to the age of all persons receiving an ambulance in Copenhagen in 2018. The median age was 83 years (IQR 75–90) compared to 64 years (IQR 41–78) in the population of all patients receiving an ambulance

When comparing the median age of the study population (83 years) t was found that these were older than the total population of patients transported in an ambulance (64 years) (Fig. 2).

Ambulances were dispatched 147 (33.8%) times from calls to 1–1–2, 87 (20.0%) from the 1813-Medical Helpline and 201 (46.2%) times at the request by GPs, hospitals, or police, where the general distribution is 50.8%, 22.3%, and 26.9% respectively. The distribution of causes for dispatch were 349 (80.2%) concerning medical issues, 83 (19.1%) injury and three (0.7%) psychiatric (Table 1).

**Table 1:** Characteristics of ambulance dispatches to patients with 48 h mortality brought to hospital non-emergently

	N	Percentage
Gender		
Female	230	52.9
Male	205	47.1
Requestor		
1–1–2	147	33.8
1813—medical helpline	87	20.0
Other	201	46.2
Call criteria		
Medical problem	349	80.2
Injury	83	19.1
Psychiatry	3	0.7
Ambulance response priority to patient		
A	143	32.9
B	285	65.5
C	7	1.6
Primary hospital admission diagnosis by ICD-10 chapter		
Respiratory	131	30.1
Abnormal signs and symptoms	77	17.7
Cardiovascular	45	10.3
Gastrointestinal	36	8.3
Infectious and parasitic	35	8.0
Trauma	34	7.8
Other healthcare contacts	25	5.7
Neoplasms	13	3.0
Endocrine and metabolic	9	2.1
Urogenital	9	2.1
Poisoning	4	0.9
Hematology	3	0.7
Neurology	3	0.7
Orthopedic	3	0.7
Psychiatry	3	0.7
No diagnose	5	1.1

The EMTs’ working diagnoses corresponded to the hospital admission diagnosis in 270 (62.1%) cases, in 129 (29.7%) it did not, and 11 (2.5%) patients were not taken to hospital.

We registered an unspecific or faulty admission diagnosis which was not possible to categorize with an organ system or condition for 25 (5.7%) patients (Table 2). A few examples are: “Z03.9 Observation for suspected disease or condition, unspecified”, “Z04.9 Examination and observation for unspecified reason”, “Z50.8 Care involving use of other rehabilitation procedures”, “Z51.5 Palliative care”, and “Z71.9 Counselling, unspecified”. Eleven patients had primary diagnoses as “death” or “cardiac/respiratory arrest” even though they were delivered alive at the hospital.

**Table 2:** Did EMS prehospital working diagnosis correspond to the patient’s primary admission diagnosis?

Selected admission diagnoses by ICD-10 chapter	Yes n (%)	No n (%)	Unknown^a^ n (%)	Total
Respiratory	93 (71.0)	35 (26.7)	3 (2.3)	131
Abnormal signs and symptoms	57 (74.0)	11 (14.3)	9 (11.7)	77
Cardiovascular	11 (24.4)	30 (66.7)	4 (8.9)	45
Gastrointestinal	23 (63.9)	13 (36.1)	0	36
Infection	18 (51.4)	16 (45.7)	1 (2.9)	35
Trauma	31 (91.2)	1 (2.9)	2 (5.9)	34
Other contacts	12 (48.0)	2 (8.0)	11 (44.0)	25

^a^Patients not transported or with an unclear admission diagnosis

When registering warning signs, it was documented in the record that 166 (38.2%) patients had dyspnea, 91 (20.9%) had some degree of affected level of consciousness, 29 (6.7%) experienced dizziness, 16 (3.7%) had had momentarily loss of consciousness, and 100 (23.0%) patients showed no warning signs.

**Fig. 3:**
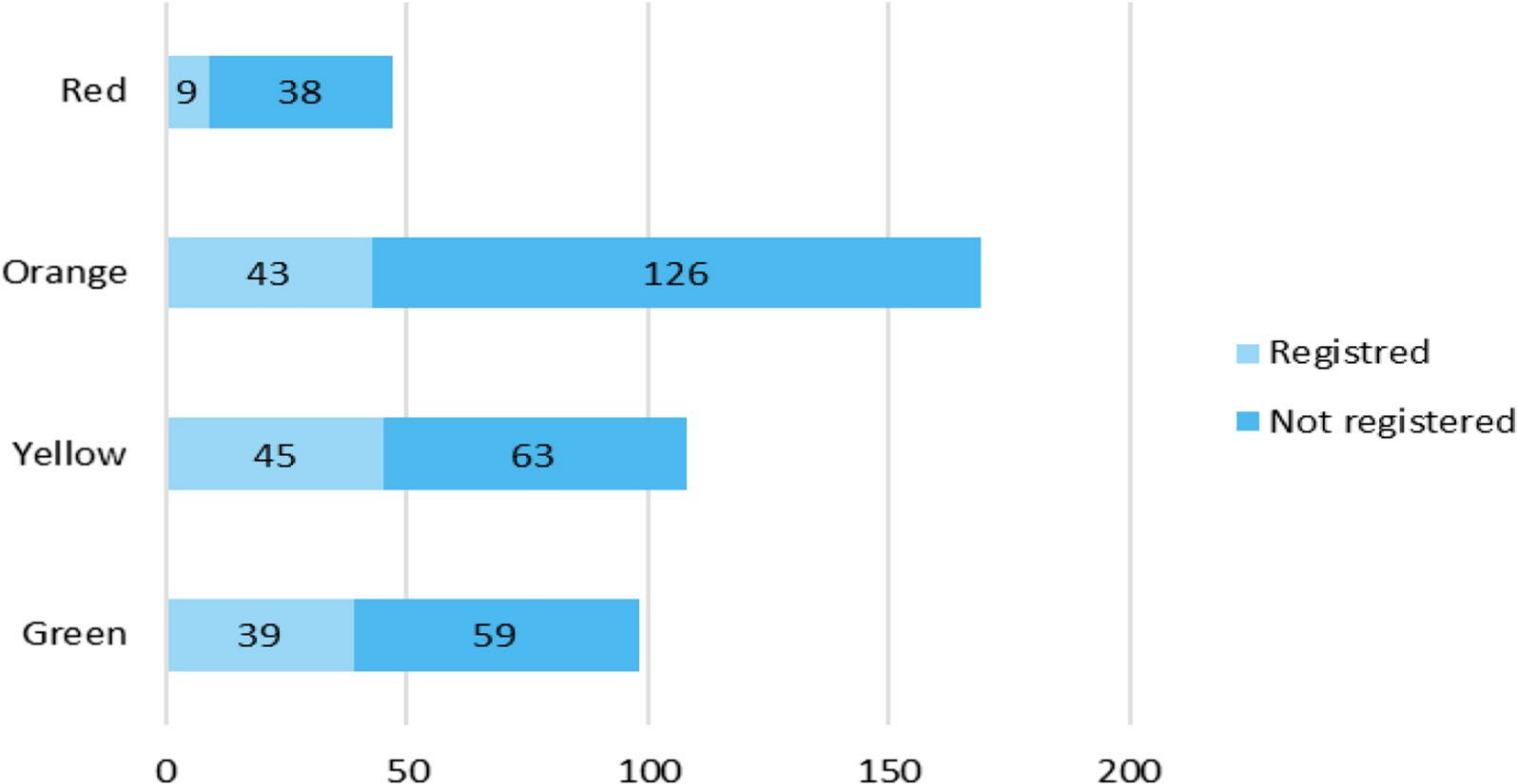
Prehospital triage category by vital values. Registered/not registered. Triage category is calculated from vital parameters. Registered categories (Light blue) have been calculated automatically by ambulance personnel. Not registered categories (Dark blue), the vital parameters are collected, but a triage category has not been assigned, and have retrospectively been calculated

In 136 (31.3%) incidents, the ambulance crew assigned a triage category prehospitally. Of the 286 patients who were not triaged 38 (13.3%) would have been triaged red and 126 (44.1%) orange based on the measured vitals alone. In total 169 (38.9%) patients met the triage criteria as orange, 108 (24.8%) yellow, 98 (22.5%) green and 47 (10.8%) red. For 13 (3.0%) patients there was insufficient data to indicate a triage degree (Fig. 3).

One or more frailty factors, other than advanced age, were documented for 192 (44.1%) patients. The primary factors were 127 (29.2%) patients noted as nursing home residents and 54 (12.4%) suffering from dementia. Other described points of frailty were use of home oxygen (n = 19, 4.4%), bedridden (n = 18, 2.1%), alcohol abuse (n = 14, 3.2%), and wheelchair user (n = 13, 3.0%). We noted previous known medical conditions for 374 (86.0%) patients. These consisted of 126 (29%) with lung disease, which primarily covered COPD, asthma, and cancer, 47 (10.8%) patients had an existing heart condition, 40 (9.2%) had diabetes, and 25 (5.7%) had a previous stroke. For 40 (9.2%) patients there was no registrations in the PPR and 21 (4.8%) were registered as not having preexisting medical conditions.

During reviewing the PPRs it was estimated that death within 48 h could not have been avoided by different treatment or prehospital visitation for 409 (94.0%) patients. For the remaining 26 (6.0%) patients, it was assessed that there possibly could have been made a difference, primarily if the patient was examined or triaged differently. A single unrecognized STEMI could possibly have been detected prehospitally if an ECG had been taken or troponins measured in a blood sample.

Of nine (2%) patients with hospital admission diagnoses regarding aortic dissection or aneurysm, none were brought to hospital under this suspicion. The working diagnoses were distributed as three patients with ‘cardiac problem’ and one as 'general weakness', 'fall', 'headache’, 'kidney stone', 'constipation' and 'syncope' respectively.

During review of the ambulance records, it was assessed that in six cases death could possibly have been avoided if the patient had been referred to a hospital with vascular surgery capacity, one case as "Probably, if referred differently " and two as "No".

A total of 27 patients of the full cohort of individuals who died within 48 h of contact with the EMS (n = 2279) had admission diagnoses regarding aortic disease, of these 15 (55.6%) were brought to hospital priority A and three (11.1%) were pronounced dead prehospitally.

## Discussion

Two thirds (n = 299, 68.7%) of the patients were not assigned a triage category prehospitally. These patients would also more often meet the criteria for orange or red triage compared to the patients who were triaged. In total half the patients (n = 216, 49.7%) triaged orange or red based on recorded vital values. The assessment deemed for 409 (94.0%) patients was that treatment received in the ambulance was adequate, and death within 48 h could not have been avoided by different treatment or triage prehospitally. We found that 270 (62.1%) of the EMTs’ working diagnoses corresponded well with the primary admission diagnosis. About halfway through reviewing the journals, a pattern emerged, that many of the times the question "Could death within 48 h have been avoided prehospitally" was answered ‘possibly’ (n = 26), the call was to acute aortic syndrome (n = 7, 26.9%). For two patients it was not estimated that death could have been avoided. It turned out that none of these nine patients with acute aortic syndrome were taken to hospital with suspected aortic disease. The prehospital personnel involved were EMTs, paramedics and prehospital physicians alike.

### Triage

One reason for the low number of prehospitally triaged patients could be due to short transport time and that the EMTs are occupied with examinations and treatment, the PPR often is completed after the patient has been handed over at the hospital, and it therefore does not seem necessary to register the triage because it primarily aims to prepare the receiving ward for the patient's condition. The fact that there is a discrepancy in the distribution of triage degree between registered and not registered patients, might be because the more stable patients require less hands-on from the EMTs and there therefore is more time to fill in the PPR en-route. When the EMT/PM actively decides not to register an orange or red triage, it could be because they are not driving to the hospital with the highest assigned priority (A), or if already close to a hospital, with an old and comorbid unstable patient, it was decided that the few minutes saved driving with highest assigned priority, would not outweigh the risk for traffic accidents and/or better the patients outcome anyway. In this case when the patient actually has been assessed as unstable prehospitally, the call would not meet any of the exclusion criteria and thus be included in the study population. One should also bear in mind that fast driving is far from the only intervention done for an unstable patient in the ambulance.

We found that 270 (62.1%) of the EMTs’ working diagnoses corresponded with the primary admission diagnosis. However, some hospital admission diagnoses did not seem valid. This could possibly be due to differences in the time which it was assigned, which could be immediately at admission or if/when the patient was moved to another ward. Eleven patients had primary diagnoses as *dead* or *cardiac/respiratory arrest* even though they were delivered alive at the hospital, which supports the claim that there could be a significant amount of time between admission and the assigned final primary diagnosis. Emergency physicians diagnostic accuracy are described in several publications [27–31]. However, the accuracy is described for very specific symptoms and conditions as stroke, cardiac conditions or trauma, and not comparably to our findings due to our population being all conditions, though selected on base of urgency of response combined with 48 h mortality.

### Avoidable deaths

The assessment deemed for 409 (94.0%) patients that treatment was adequate, and death within 48 h could not have been avoided by different treatment or triage prehospitally, as the patients were generally old (median age 83 years) and frail (127 (29.2%) were registered as nursing home residents and 54 (12.4%) as suffering from dementia).

For the remaining 26 (6.0%) patients, it was assessed that there possibly could have been made a difference, primarily based on the assumption that the patient, if recognized as being unstable, would have received faster relevant treatment^5^. The assessment included a general evaluation of standard of care, how paramedics addressed and responded to patients documented vital signs and symptoms, and if medication was given according to standards. We do however not know how the handover of the patient to the receiving ward went, and if the call was made. This assessment was solely based on the available dispatch message and notes in the PPR. This is a retrospective study, and therefore the quality of the registrations in the available medical records are paramount. We do not know if there has been signs or symptoms not recorded. So, this assessment could very well be different if an alternative set of parameters were used upon the medical records.

### Aortic aneurysm and dissection

About halfway through reviewing the journals, a pattern emerged, that many of the times the question "Could death within 48 h have been avoided prehospitally" was answered ‘possibly’ (n = 26) the call was to acute aortic syndrome (n = 7, 26.9%), for two patients it was not estimated that death could have been avoided. It turned out that none of these nine patients with acute aortic syndrome were taken to hospital with suspected aortic disease. The prehospital personnel involved were EMTs, paramedics and physicians alike.

Aortic aneurysms and dissections are time critical conditions that must be brought to the university hospital with upper aortic repair facility for vascular surgery, it is therefore important they are recognized prehospitally. The nine patients constituted 33.3% of totally 27 patients with admission diagnoses regarding acute aortic syndrome in the full cohort of individuals who died within 48 h of contact with the EMS in 2018. For the remaining 18 patients we have not registered a prehospital working diagnosis, but 15 were brought to hospital priority A and three were pronounced dead prehospitally, although this number may be greater as patients pronounced dead prehospitally do not usually receive an admission diagnosis. Yamashita et al. [32] have studied the quality of EMS assessment of acute aortic syndrome. They found that EMS correctly assessed the risk for acute aortic syndrome in 195 (54.0%) of 361 patients, and that it was less frequently detected in the elderly with dyspnea and syncope/faintness.

### Strengths of this study

In Denmark, we have comprehensive public registers of all citizens' contacts with the health services. This provides a large cohort, as well as avoids selection bias [20].

All ambulance dispatches in Copenhagen in 2018 were included.

### Limitations in this study

Retrospective studies by nature have some limitations when dealing with medical records. The patient record is produced when the clinical personnel is busy with patient care and transport (or immediately thereafter), and for this reason the purpose of the recordings are aimed to provide information necessary for other personnel involved in the handling of the patient. The record is not written to support questions, aims or purposes of research. The quality of data was very dependent on the EMT’s/PM’s completion of the PPR, as well as whether there had been an error in the data connection to PPR. We found that 40 (7.2%) of 555 records were without content.

Recorded measured vital parameters may be subject to some uncertainty [33], especially SpO2 is dependent on the patient's peripheral perfusion, and the blood pressure measurement can vary greatly when taken in a moving vehicle. Counting the respiratory rate is probably rarely done conscientiously over one minute, but rather estimated with consequent misjudgments [34]. Automatic registration of respiratory rate using EtCO_2_ measurement or ECG monitoring in the prehospital setting, with the current technology applied is practically useless, as it is extremely sensitive to the patient moving, talking, coughing, or being in a moving vehicle etc. It was therefore necessary to make a subjective assessment of whether the measured vital values were low, normal, or high based on trends in the values and notes in the PPR.

The answers in the questionnaire were based on primary investigator’s subjective assessment of the registered data when reviewing the records. Certain points had to be estimated when not specifically recorded, e.g. working diagnosis based on dispatch information, measured vital parameters and notes.

Negative findings are not always registered in the PPR, just as it not always is noted whether the patient is a wheelchair user or bedridden. The use of home oxygen could be determined somewhat more precisely from the field 'Treatment before arrival' in the PPR where there is the possibility to tick 'oxygen'.

A more reliable cause of death could maybe be retrieved from the Danish Registry of Causes of Deaths [35], but we had no access to this database.

## Conclusions

We found that the study population of patients who died within 48 h after being treated on scene or taken to hospital non-emergently by ambulance in Copenhagen in 2018 were of high age (median 83 years) and that 86.0% of the patients had one or more serious pre-existing medical conditions.

For 94.0% of the patients it was not estimated that death within 48 h could have been avoided by different treatment or visitation. The prehospital providers were less likely to register a triage color before arrival to hospital, when the patient met the criteria for the categories Red and Orange [8] based on the measured vitals.

Acute aortic syndrome was not identified in nine (33.3%) of 27 patients, with delayed definitive and potentially life-saving treatment as a result.

## Supplementary Information

**Additional file 1.** Questionnaire used for reviewing the Prehospital Patient Records.

## Data Availability

Data is available on reasonable request. Please email the corresponding author to request the relevant data. Please provide the authors of the article with a detailed protocol for the proposed study and supply information about the funding and resources to conduct the study. If appropriate, invite the original author(s) to participate in the reanalysis.

## Abbreviations

COPD: Chronic obstructive pulmonary disease
CPR: Central person register
ECG: Electrocardiogram
EMCC: Emergency medical communications center
EMS: Emergency medical service
EMT: Emergency medical technician
EtCO_2_: End-tidal CO_2_
GP: General practitioner
MCCU: Mobile critical care unit
PM: Paramedic
PPR: Prehospital patient record
STEMI: ST-elevation myocardial infarction
