# Supplementary material for: Characteristics of low acuity prehospital emergency patients with 48-h mortality, an observational cohort study
Source: Scand J Trauma Resusc Emerg Med. 2022 Dec 8;30:64. doi: 10.1186/s13049-022-01048-8 (PMC9730555; doi:10.1186/s13049-022-01048-8)
Supplement: Supplementary file 1 — Additional file 1. Questionnaire used for reviewing the Prehospital Patient Records. [file 13049_2022_1048_MOESM1_ESM.docx]

**Additional file 1: Appendix**

Questionnaire used for reviewing the Prehospital Patient Records

1. WorkSetId ______________________________
2. Prior medical conditions

- Not registered
- Unknown
- None
- Heart
- Lung
- Stroke
- Seizures / epilepsy
- Diabetes
- Psychiatry
- Other ____________________________

1. Primary assessment

|  | Unaffected | Slightly affected | Very affected | Not registered |
| --- | --- | --- | --- | --- |
| A | € | € | € | € |
| B | € | € | € | € |
| C | € | € | € | € |
| D | € | € | € | € |

1. Secondary assessment

|  | Unaffected | Slightly affected | Very affected | Not registered |
| --- | --- | --- | --- | --- |
| A | € | € | € | € |
| B | € | € | € | € |
| C | € | € | € | € |
| D | € | € | € | € |

1. Treatment

- None
- O_2_ binasal
- O_2_ mask
- IV catheter
- IV catheter, attempted
- Fluids, iv
- Salbutamol, inhalation
- Fentanyl, iv
- Other ________________________

1. Vitals

|  | Low | Normal | High | Not registered |
| --- | --- | --- | --- | --- |
| Respirations | € | € | € | € |
| SpO_2_ | € | € | € | € |
| Heart rate | € | € | € | € |
| Blood pressure | € | € | € | € |
| GCS | € | € | € | € |
| Blood glucose | € | € | € | € |
| Temperature | € | € | € | € |

1. Triage

|  | **Red** | **Orange** | **Yellow** | **Green** |
| --- | --- | --- | --- | --- |
| **A** | Threatened airway |  |  |  |
|  | Inspiratory stridor |  |  |  |
| **B** | SpO_2_ < 80 % without O_2_ | SpO_2_ < 90 % without O_2_ | SpO_2_ < 95 % without O_2_ | SpO_2_ ≥ 95 % without O_2_ |
|  | SpO_2_ < 90 % with O_2_ | SpO_2_ < 95 % with O_2_ |  |  |
|  | RR > 35 or < 8 | RR > 30 | RR > 25 | RR: 8-25 |
| **C** | HR > 140 | HR > 120 or < 40 | HR > 110 or < 50 | HR: 50 - 110 |
|  | BPsys < 80 mmHg | BPsys < 90 mmHg |  | BPsys ≥ 90 mmHg |
| **D** | GCS ≤ 8 | GCS 9 - 13 | GCS = 14 | GCS = 15 |
| **E** | Temp < 32 °C | Temp > 40 °C or 32-34 °C | Temp > 38 °C or < 35 °C | Temp: 35 – 38 °C |

- Red
- Orange
- Yellow
- Green
- Not registered

1. ECG
   - Not registered

- Sinus rhythm
- Atrial fibrillation
- STEMI
- Pace rhythm
- Broad QRS
- Other _______________________________

1. Warning signs

- Not registered
- None
- Dyspnea
- Bleeding
- Syncopy
- Vertigo
- Depressed consciousness
- Seizures
- Other _________________________________

1. Pain

- Not registered
- None
- Head
- Chest
- Abdomen
- Back
- Arms
- Legs
- Other __________________________________

1. Symptom debut

- Hours
- Days
- Weeks
- Unknown
- Other __________________________________

1. Prehospital working diagnosis ______________________________________________
2. Does working diagnosis correspond to admission diagnosis?

- Yes
- No
- Unclear / No admission diagnosis
- Not brought to hospital
- Other __________________________________

1. Frailty

- Dementia
- Alcoholism
- Nursing home resident
- Bedridden
- Wheelchair user
- Not brought to hospital
- Home oxygen
- None / unclear
- Other __________________________________

1. Could death within 48 hours have been avoided prehospitally?

- No
- Yes
- Other __________________________________

1. Could death within 48 hours have been predicted prehospitally?

- No
- Yes
- Unclear
- Other __________________________________

1. Quality of the record

- Adequate
- Lacking
- Empty

1. Comments ____________________________________________________________________________________________________________________________________________________
2. Save for later study

- Yes
- No, poor quality
